# Impact of post-match fatigue on peak force in elite youth soccer players: Analysis of 48 to 72 hours post-match using the isometric mid-thigh pull exercise

**DOI:** 10.5114/biolsport.2025.150044

**Published:** 2025-04-28

**Authors:** Ricardo Pimenta, Lúcio Cunha, Fábio Yuzo Nakamura

**Affiliations:** 1 Research Center of the Polytechnic Institute of Maia (N2i), Maia Polytechnic Institute (IPMAIA), Castêlo da Maia, 4475-690 Maia, Portugal; 2 Department of Rehabilitation and Optimization of Performance (DROP), Futebol Clube Famalicão – Futebol SAD, Famalicão, Portugal; 3 CIPER, Faculdade de Motricidade Humana, Universidade de Lisboa, Cruz Quebrada Dafundo, Portugal; 4 Porto Biomechanics Laboratory, Faculty of Sport, University of Porto, Porto, Portugal; 5 FSI Lab, Football Science Institute, Granada, Spain; 6 Research Center in Sports Sciences, Health Sciences and Human Development (CIDESD), University of Maia, Maia, Portugal

**Keywords:** Neuromuscular fatigue, Muscle damage, Isometric, Soccer, Performance, Recovery

## Abstract

This study aimed to evaluate the time-course of the recovery of force-generating capacity in young adult soccer players post-match. Seventy-three Under-19 and Under-23 soccer players were assessed using the isometric mid-thigh pull (IMTP) test. Measurements were taken during a baseline session (under non-fatigued conditions) and at 48 h and 72 h post-match. External and internal load metrics were recorded on match day and training days using a Global Positioning System and the session session-rating of perceived exertion, respectively. For each game, players’ observations were divided into experimental and control conditions, reflecting playing more than 60 minutes and 0 minutes of match participation, respectively. Within-subject correlations between selected external match load metrics and internal load, and changes in IMTP peak force (PF), were analyzed for players in the experimental condition. The results demonstrated that, in the experimental condition, the IMTP PF was impaired by 8.1% at 48 h post-match (p < 0.001, d = 0.72) and 6.2% at 72 h post-match (p < 0.001, d = 0.68), with no significant differences between 48 h and 72 h post-match (p > 0.05). However, no correlations were observed between changes in IMTP PF at 48 h and 72 h post-match and either external or internal load. Moreover, an 8.2% reduction in IMTP peak force from baseline to 72 h post-match (1603N vs 1471N, p = 0.01, d = 0.46) was observed in the control condition, which can be attributed to the load on MD+2. This finding suggests that the IMTP is a sensitive test for detecting fluctuations in the recovery of force-generating capacity, further highlighting that soccer players do not achieve full recovery in this test within the specified time frame.

## INTRODUCTION

Fatigue is associated with performance decrements, which have often been suggested as a potential contributing factor to injury [1]. Fatigue is characterized as an inability to maintain a given exercise intensity or power output, resulting from either acute physical exertion or residual effects (i.e., inadequate recovery from repeated exposures to load) [2]. Acute fatigue, which develops during and immediately after physical activity, is attributed to a combination of central and peripheral fatigue mechanisms [2]. Central fatigue impairs voluntary muscle activation and primarily occurs after submaximal, low-intensity muscle contractions [3]. Peripheral fatigue refers to an impaired capacity for muscle contraction and can be induced by disturbances in action potential propagation, excitation-contraction coupling, and mechanisms of contractile force production [3]. In soccer, the interaction between central and peripheral fatigue leads to performance decrements that may persist for several days postmatch, with varying recovery profiles observed across distinct physical capacities. Indeed, the contribution of muscle damage to these performance decrements must be considered; previous reports have highlighted the sustained elevation of muscle damage markers, such as creatine kinase and fiber disruptions, up to 72 hours post-match in young and amateur soccer players [4, 5]. Performance impairment in the first days following a match has been previously reported in jumping and sprinting abilities [6] and in different resistance exercises, such as the hip-thrust and half-squat [7]. Notably, one study observed that maximal force production of the knee extensors took 72 h to recover post-match [8]. Understanding the time-course of recovery for these variables provides valuable insights to inform decision-making during microcycles, helping to minimize fatigue and the development of muscle damage before the subsequent match.

Moreover, as aforementioned, there is an established epidemiological association between acute fatigue and injury in soccer [9]. Indeed, studies have reported neuromuscular impairments in professional soccer players following matches [8, 10]. Changes in the neuromuscular system can result from muscle fatigue and muscle damage, which can be evaluated using parameters such as peak force (PF), a measure of force-generating capacity [11]. The IMTP is a relatively recent exercise method to assess strength and neuromuscular fatigue in athletes [12, 13]. One of the advantages of the IMTP, compared to single-joint isometric tests of strength, is that it is generally highly associated with dynamic exercise performance [14, 15]. Significant correlations between IMTP peak force and performance in several physical tests, such as the 5-meter sprint [16], 10-meter sprint [17], 20-meter sprint [18], and changeof-direction tests [15, 17], have been reported. Notably, moderate to strong negative correlations have been identified between delayedonset muscle soreness,which is associated with muscle damage, and CMJ height in youth soccer players up to MD+2 [19]. Similarly, strong negative correlations were found between delayed-onset muscle soreness and IMTP values in professional soccer players up to MD+2 and MD+3 [20]. Moreover, a previous study reported that fatigue and muscle damage persist up to 72 hours after a match, indicating decreases in isometric strength test values for the hamstrings and muscle fiber disruptions within this timeframe in amateur soccer players [5]. However, the hamstrings represent only one muscle group activated in soccer, and the IMTP test reflects contributions from multiple muscles. Furthermore, it is unclear whether differences in recovery between 48 and 72 hours post-match occur in young soccer players, as well as whether a correlation exists between a neuromuscular parameter and match-related Global Positioning System (GPS) external load. Such insights would provide valuable information for coaches to plan training stimuli. A higher frequency of repetitive braking cycles during the match is likely to increase fatigue and injury risk [21], as these actions are associated with a high eccentric force demand [22], which, in turn, significantly contributes to muscle damage [23, 24].

The aim of the present study was to: (i) compare match-related recovery between players with high participation in the match and those who did not play, (ii) compare IMTP PF at 48 h and 72 h postmatch with baseline in the experimental condition, and (iii) examine the relationship between external and internal match load and changes in IMTP PF in elite young soccer players who played more than 60 min. We hypothesized that: (i) IMTP PF would differ between players with different match participation at 48 h post-match, (ii) a noticeable decrease in force-generating capacity would occur at 48 and 72 hours post-match compared to baseline in the experimental condition, and (iii) a significant correlation would exist between PF and the number of decelerations during the match.

## MATERIALS AND METHODS

### Participants

Seventy-three elite young adult soccer players (age: 18.6 ± 1.3 years, weight: 72.7 ± 6.5 kg, height: 179.4 ± 6.2 cm) from two teams competing in the Under-19 and Under-23 Portuguese National Championship (highest national division for these specific age groups) were invited to participate in this study. For each game, the players were divided into two conditions: a control condition, in which the players did not participate at all on match day (MD), and an experimental condition, in which players participated for at least 60 minutes during the match. This division resulted in 170 observations for the experimental condition and 28 for the control condition. The observations associated with player participation of less than 60 minutes but more than 0 minutes during the matches were not included in the study (n = 90). We have classified players as elite because 22 of them played in the Youth Champions League (Tier 4, according to McKay et al., 2022), while the remaining players were classified as Tier 3 (national level) [25]. All participants read and signed an informed consent form before participating in the study. The local Ethics Committee approved the study (#62/2022).

### Protocol

Participants performed three preliminary sessions to become familiar with the equipment for the maximum voluntary isometric contraction (MVIC) evaluation during the IMTP exercise. The testing sessions were divided into one baseline session to record the maximal PF for each athlete and sessions conducted at 48 h and 72 h post-match. Between one and seven IMTP tests at 48 h and 72 h post-match were analyzed per player. To minimize a possible fatigue effect, the baseline session was performed after four days of rest. The entire nutritional regimen is standardized, as all players have an individual nutritional plan provided by the nutritionist, with daily monitoring of their weight and weekly assessments of hydration status and skinfold measurements. All tests were conducted at the same time of day before the soccer training session and followed the same warm-up protocol. On testing days, participants began with 5 minutes of cycling, followed by 5 minutes of mobility exercises under the guidance of a strength and conditioning coach. After warming up, participants were positioned in a standing position, with the bar height adjusted to ensure the athlete achieved optimal knee (125–145°) and hip (140–150°) angles [26]. Then, participants performed two repetitions, **f**ollowing instructions to generate maximum force as quickly as possible.

On MD, players who did not participate in the game (control condition) performed a compensatory post-match training session immediately after the match. On MD+2 (48 hours post-match), players who participated for more than 60 minutes (experimental condition) engaged exclusively in recovery protocols (e.g., standardized stretching sessions and the use of compression boots for all players) or performed low-intensity recreational exercises on the field, while the control condition performed standard field training using smallsided games such as 1 vs 1 (with an area of approximately 10 m^2^) and 5 vs 5 + goalkeepers, utilizing the penalty area and two 11-aside football goalposts.

### Data processing

The force was measured at a sampling rate of 80 Hz using custom-made equipment. For each session, the highest PF of the two MVIC trials was considered for Δ IMTP analysis. The Δ IMTP was calculated as a pre-to-post change:

Δ IMTP=(PF at 48 h/72 h)−(Baseline PF)/(Baseline PF)

External training and match load were monitored using a portable 10 Hz GPS device (Catapult Vector S7, Catapult Sports, Melbourne, Australia), and internal load was monitored using the session-rating of perceived exertion (s-RPE) [27]. To guarantee higher data reliability and reduce inter-unit error, each player consistently used the same GPS unit throughout all data collections. GPS data from matches were downloaded using the manufacturer’s software (Catapult Openfield, version 3.10, Firmware 8.1). The following variables were collected: total distance covered while running (m/min, 14.4 km/h–19.7 km/h), high-speed running (HSR, m/min, > 19.8 km/h – 25.2 km/h), sprinting distance (m/min, > 25.2 km/h), number of high accelerations (ACC, > 3 m/s^2^) and high decelerations (DEC, < -3 m/s^2^).

### Statistical analysis

All statistical analyses were performed using R software (version 2023.03.1+446, R Foundation for Statistical Computing, Vienna, Austria) and figures were created using GraphPad Prism 10 (GraphPad Software, San Diego, CA, USA) and R software. Prior to statistical analysis, data were checked for normality through visual inspection of Q-Q plots and histograms, and objectively via the Kolmogorov-Smirnov test. All external and internal load metrics showed a non-normal distribution (p < 0.05), so non-parametric tests were used. Differences between intervention and control conditions in MD+2 training loads were analyzed using the Mann-Whitney U test.

A linear mixed model analysis was used to analyze the differences between baseline, 48 h and 72 h post-match IMTP peak force in both experimental (> 60 minutes) and control condition (0 minutes). Models were compared using the performance package. All models were estimated via Restricted Maximum Likelihood (REML), and model appropriateness was verified by examining the QQ-plots of the residuals. Thus, the variable time (baseline, post-48 h, and post-72), condition (experimental, control), and an interaction term were included as fixed effects, and player ID was included as a random effect. When significance was observed for the main effects, *post-hoc* pairwise comparison tests using the Tukey correction were computed to assess differences (i.e., IMTP PF between times and conditions) using the emmeans package. In order to quantify the effect size (ES) of the *post-hoc* comparisons, Cohen’s *d* was calculated by converting the t statistics to *d* using the effect size package [28]. The magnitude of the ES was interpreted as follows: trivial (*d* < 0.2), small (*d* = 0.20–0.59), moderate (*d* = 0.6–1.19), large (*d* = 1.2–2.0), very large (*d* > 2.0) [29]. The significance level was set as p ≤ 0.05 for all statistical comparisons.

Within-subjects correlations (r, 95% CI) [30] were tested between external load metrics (total distance, high-speed running distance, sprint distance, ACC > 3 m/s^2^, DEC < -3 m/s^2^), internal load (s-RPE) and ΔIMTP PF at 48 h and 72 h post-match, only for players with more than 60 minutes of match participation. We qualitatively interpreted the magnitudes of correlation using the following criteria: trivial (r ≤ 0.1), small (r = 0.1–0.3), moderate (r = 0.3–0.5), large (r = 0.5–0.7), very large (r = 0.7–0.9), and almost perfect (r ≥ 0.9) [29]. When the 95% CI overlapped positive and negative values, the effect was deemed unclear.

## RESULTS

Match day external load metrics are presented in Table 1 (experimental condition) and Table 2 (control condition). Regarding the external and internal training loads on MD+2, there were significant differences between the experimental and control conditions, favoring the latter in all metrics. Table 3 shows the mean ± standard deviation for the external and internal load values during the postmatch recovery session (experimental condition) and the standard field training using small-sided games (control condition).

**TABLE 1:** External and internal match’s loads for the experimental condition (> 60 min).

Match’s External and Internal Loads	
Duration (min)	91 ± 15
Total distance (m)	9511 ± 1470
High speed running distance (m)	529 ± 175
Sprint distance (m)	96 ± 58
Accelerations > 3 m/s^2^ (n)	19 ± 12
Decelerations < -3 m/s^2^ (n)	25 ± 13
s-RPE (AU)	731 ± 203

Legend: Data are mean ± standard deviation; min, minutes; m, meters; n, number of events; s-RPE, session rating of perceived exertion; AU, arbitrary units.

**TABLE 2:** External loads of compensatory post-match training of the control condition (0 min).

Compensatory Post-Match Training Loads	
Duration (min)	10 ± 4
Total distance (m)	1058 ± 273
High speed running distance (m)	235 ± 142
Sprint distance (m)	26 ± 40
Accelerations > 3 m/s^2^ (n)	18 ± 6
Decelerations < -3 m/s^2^ (n)	15 ± 5

Legend: Data are mean ± standard deviation; min, minutes; m, meters; n, number of events.

**TABLE 3:** External and internal loads at MD+2 training session for the experimental condition (> 60 min) and control condition (0 min).

	Experimental	Control	p-value
Duration (min)	45 ± 11	59 ± 11	< 0.001
Total distance (m)	2249 ± 509	3570 ± 616	< 0.001
High speed running distance (m)	6 ± 12	78 ± 94	< 0.001
Sprint distance (m)	0 ± 0	4 ± 11	< 0.001
Accelerations > 3 m/s^2^ (n)	3 ± 4	13 ± 7	< 0.001
Decelerations < -3 m/s^2^ (n)	1 ± 2	9 ± 6	< 0.001
s-RPE (AU)	86 ± 43	186 ± 82	< 0.001

Legend: Data are mean ± standard deviation; min, minutes; m, meters; n, number of events; s-RPE, session rating of perceived exertion; AU, arbitray units.

### Comparisons of IMTP PF at baseline, 48 h and 72 h post-match

**FIG. 1:**
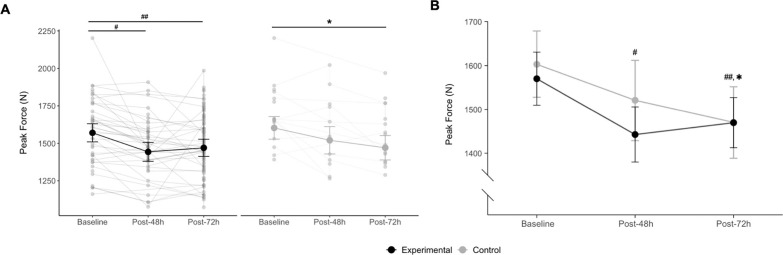
(A) Inter individual and (B) average values of isometric mid-thigh pull Peak Force at baseline, 48 h, and 72 h post-match. Experimental condition: # significantly different from baseline (p < 0.001), ## significantly different from baseline (p < 0.001); Control condition:* significantly different from baseline (p = 0.01). Data are marginal means and 95% confidence intervals.

There was no interaction (p = 0.29) between the two fixed effect factors (time and condition), while a main effect of time was observed for IMTP peak force (p < 0.001). In the experimental condition, *post-hoc* comparisons showed an 8.1% moderate decrease in IMTP PF from baseline to 48 h post-match (1570 N vs. 1443 N, p < 0.001, *d* = 0.72) and a 6.2% moderate decrease from baseline to 72 h postmatch (1570 N vs. 1470 N, p < 0.001, *d* = 0.68), with no significant differences between 48 h and 72 h post-match (p = 1.00). In the control condition, *post-hoc* comparisons revealed an 8.2% small reduction in IMTP peak force from baseline to 72 h post-match (1603 N vs. 1471 N, p = 0.01, *d* = 0.46). Figure 1 illustrates the differences in IMTP PF at baseline, 48 h, and 72 h post-match in both conditions.

### Repeated measures correlations between ΔIMTP at 48 h and 72 h post-match and external and internal load variables

Table 4 shows the results of the repeated measures correlations between ΔIMTP peak force at 48 h and 72 h post-match and external and internal load variables. No significant correlations were found between external or internal load variables and ΔIMTP peak force at 48 h and 72 h post-match (p > 0.05).

**TABLE 4:** Within-subject correlations between external load metrics, session rate perceived exertion (s-RPE) and ΔIsometric Mid Thigh Pull Peak Force (IMTP PF) at 48 h and 72 h post-match.

	Δ IMTP PF 48 h post-match			Δ IMTP PF 72 h post-match		

	r (95C% CI)	p-value	Description	r (95C% CI)	p-value	Description
**External loads metrics**						
Total distance	0.02 (-0.40–0.44)	0.92	Unclear	0.04 (-0.19–0.26)	0.75	Unclear
High speed running distance	0.05 (-0.37–0.47)	0.80	Unclear	-0.07 (-0.29–0.16)	0.55	Unclear
Sprint distance	-0.01 (-0.43–0.42)	0.98	Unclear	0.02 (-0.21–0.24)	0.86	Unclear
Accelerations > 3 m/s^2^	-0.33 (-0.66–0.11)	0.13	Unclear	-0.16 (-0.37–0.07)	0.17	Unclear
Decelerations < -3 m/s^2^	-0.41 (-0.71–0.01)	0.06	Unclear	-0.03 (-0.26–0.20)	0.79	Unclear

**Internal loads**						
s-RPE	-0.15(-0.56–0.31)	0.52	Unclear	-0.10 (-0.32– 0.13)	0.39	Unclear

Legend: Data are mean ± standard deviation; s-RPE, session rating of perceived exertion.

## DISCUSSION

This study examined the time-course of force-generating capacity after a soccer match, measured at 48 h and 72 h post-match, using IMTP PF and the relationship between this variation and selected internal and external match load measures, in Under-19 and Under-23 players competing in the Portuguese National Championship. The main findings were: (i) no differences were found on PF between conditions at 48 h and 72 h post-match; (ii) players who played > 60 min during the match exhibited a significant moderate decrease in IMTP PF at 48 h, while the decrement in the control condition was also moderate, but non-significant; (iii) IMTP is a neuromuscular test sensitive enough to detect fatigue and muscle damage effects, through PF assessment following a soccer match at 48 h and 72 h post-match, with no significant differences between the 48 h and 72 h time-points in the experimental condition and; (iv) the observed decrease in IMTP performance at 48 h and 72 h post-match (analyzed as ΔIMTP PF) was not significantly associated with any external load measures using GPS or an internal load parameter such as s-RPE in the experimental condition.

The results of the present study indicate a decrease in force-generating capacity at 48 h after a soccer match, as evidenced by decreased PF values obtained via IMTP, which remained significantly reduced at 72 h post-match in the experimental condition. Indeed, the results demonstrate that the impact of the match remains evident, with a moderate effect, during the first 72 hours (48 h: *d* = 0.72; 72 h: *d* = 0.68), which is consistent with studies documenting that a 48 h time-period may not be sufficient for the complete restoration of neuromuscular performance [4, 8, 10, 31]. Moreover, these results are consistent with a recent study reporting that fatigue persists up to 72 hours post-match, indicating not only muscle fiber disruptions within this timeframe but also decreases in isometric strength and jump test performance in amateur soccer players [5]. Interestingly, IMTP sensitivity was also demonstrated in relation to the effects of training-related factors on force-generating capacity in the control condition. More specifically, a non-significant 5.1% moderate reduction (*d* = 0.75) in IMTP PF was observed 48 h postmatch, despite 0 minutes of match participation. This reduction may be explained by the compensatory post-match training (see Table 2). In fact, Table 2 show an average volume of accelerations and decelerations similar to those observed during the match, but with half the volume of HSR. Since this volume was accumulated over a shorter period (~10 min, excluding ~5 min of the recovery time), the relative training intensity was high and may have been one of the causes of the observed decrease in force-generating capacity 48 hours after the match in the control condition. Secondly, significant differences were observed at 72 h post-match, as confirmed in Table 2, which aligns with differences in training load on MD+2 across all internal and external load measures between the experimental and control conditions. Moreover, the load in the control condition during compensatory training was higher than the load during MD+2 training, which is further supported by the fact that the reduction in PF from 48 h to 72 h post-match (3.1%) was smaller compared to the decrease from the match to 48 h post-match. Furthermore, it seems that the loading approach applied to the control players achieved the desired effect, as all players (experimental and control) began the MD+3 (72 h post-match) training session with a similar level of force-generating capacity (Figure 1-B).

The implications for strength training (particularly for the lower limbs) should be taken into account, since at 72 h post-match, players are still in the recovery phase. Given that significant underperformance in IMTP PF is maintained until 72 h post-match, it is plausible that players might not demonstrate sufficient neuromuscular readiness to cope with training stimuli that could significantly increase fatigue, potentially compromising their recovery until the next match. Although not exactly similar to our results, other authors have shown that neuromuscular parameters, such as maximal voluntary contraction of the knee extensors and squat jump height, require up to 72 h for complete recovery after a match [8, 31]. Compared to IMTP, the tests applied in the aforementioned studies likely assess distinct physical capabilities [32] and are associated with different muscle groups. Additionally, it is important to consider that only the IMTP was used in our study, which differs from jump metrics and indicators of DOMS, and this may have led to different results.

As previously mentioned, an increased frequency of repetitive braking cycles during the match is likely to accelerate the onset of fatigue [21], owing to substantial eccentric force demands [22], which significantly contribute to muscle damage [23, 24]. Furthermore, fatigue induced by the stretch-shortening cycles, which are also present in ACC/DEC efforts, may extend the time required for complete recovery of physical performance to 4–8 days [33]. Since, in tactical periodization, most of the training on 72 h post-match (MD+3) is characterized by ACC/DEC efforts [34], the presence of neuromuscular fatigue and muscle damage may negatively impact athlete performance and increase the risk of injury. Therefore, coaches should carefully consider the volume of ACC/DEC during training at 72 h post-match, as several athletes who played more than 60 minutes may still exhibit lower levels of force-generating capacity. These findings are also relevant when considering whether to train on MD+2. Previous studies have demonstrated a higher injury rate in players training on MD+2 (48 h post-match) compared to MD+1 (24 h post-match) [35]. In the present study, it was not possible to measure fatigue at 24 h (day off), since players only trained on MD+2, where the common approach is to implement a lighter session. However, this session still falls within the category of active recovery, which may potentially delay full recovery. Providing rest to players 24 h after the match rather than at 48 h may have important implications for their recovery. Nevertheless, this warrants further investigation.

Furthermore, our findings revealed no correlation between the ΔIMTP PF performance at 48 h and 72 h and any measures of external or internal load. Notably, previous studies have reported associations between IMTP PF and similar metrics under non-fatigued conditions. For instance, Townsend et al. [15] found that PF obtained during an IMTP test was significantly correlated with 20-m sprint performance. Similarly, Thomas et al. (2015) found a significant correlation between 5- and 20-m sprint times and IMTP PF in collegiate soccer and rugby players [15, 18]. Additionally, a significant correlation has been reported between performance in a change of direction test (modified 505 test) and IMTP PF [15, 17, 18]. However, in these studies, IMTP was only measured in a recovered state.

Contrary to the initial hypothesis, the results of the present study suggest that neither ACC nor DEC performance during the match is correlated with ΔIMTP PF over 72 h. Although IMTP PF is sensitive to neuromuscular fatigue and muscle damage induced by match-play, the lack of association between the PF variation and ACC/DEC metrics may indicate that the ACC/DEC performance does not influence force-generating capacity variation during the 72 h postmatch period. One possible explanation is that DEC involves an associated eccentric component that is not assessed in an isometric test. However, the aim of the study was to analyze force-generating capacity using a more pragmatic and accessible approach, without compromising the players’ performance throughout the week and on the following day (48 h vs. 72 h analysis). The isometric contraction regimen of the test guarantees a lower level of muscle damage when compared to eccentric and isometric exercises involving longer muscle lengths, resulting in a less prolonged loss of maximum force capacity [36, 37]. The absence of differences regarding the 48 h vs 72 h time points in terms of neuromuscular performance is in opposition to the findings of Brownstein et al. (2017) and Rampinini et al. (2011), who reported a complete restoration of neuromuscular performance at 72 h and 48 h post-match in semi-professional and professional soccer players, respectively [8, 10]. Nevertheless, the authors assessed the maximal voluntary contraction of knee extensors, whereas in the present study, an IMTP test was applied, involving a much wider range of muscles beyond the knee extensors. Also, the players’ competitive level differed between studies, probably having a significant impact on the time-course of neuromuscular recovery since soccer match-induced fatigue appears to be less pronounced in high-level and experienced players [10]. Indeed, as shown in Figure 1-A, the individual responses of each player to external match load are highly heterogeneous and likely depend on their physical capacities. Consequently, the present data demonstrate that player loads should be individually prescribed throughout microcycles according to each player’s specific needs as well as their fatigue and muscle damage status. This approach is thought to not only optimize adaptive responses but also minimize the risk of excessive training and the likelihood of musculoskeletal injuries. The relationship between injury and post-match neuromuscular fatigue or muscle damage after a match warrants further investigation.

This study has some limitations. Firstly, regarding the time course of force-generating capacity measurements, it was not possible to take measurements at 24 h post-match, as this was the players’ rest day within the microcycle. Additionally, measurements were not taken at 96 h due to findings from previous studies [5, 8]. Future research may benefit from assessing recovery status at these time points. Secondly, we solely used IMTP to assess neuromuscular fatigue and muscle damage effects; therefore, caution must be exercised when interpreting and generalizing these results. Finally, despite the training load being programmed and monitored by sports scientists and nutrition being supervised by a nutritionist, other factors may have influenced the results. Moreover, the findings reflect only a sample of elite young adult soccer players, with an unbalanced number of observations between the experimental and control conditions.

## CONCLUSIONS

The IMTP test is sensitive for assessing fatigue and muscle damage induced by a soccer match in young soccer players (U19 and U23). The decrease in force-generating capacity persists at 48 h and 72 h post-match, without significant differences between 48 h and 72 h time points in the experimental condition, indicating that soccer players do not fully recover at either 48 or 72 hours post-match. Although there were no significant differences between conditions at 48 h and 72 h, this could be attributed to the compensatory training immediately after the match and at 48 h post-match (MD+2). Indeed, training on MD+2 for the control condition (0 minutes) induces a decrease in the force-generating capacity of the players, allowing them to reach a similar level to the experimental condition (> 60 min) on MD+3. The Δ48 h and Δ72 h were not found to be correlated with any external or internal load, indicating that these metrics, individually, cannot directly reflect parameters modified by match load. Moreover, the IMTP exercise appears to be not only an easily applicable but also a non-time-consuming solution to assess force-generating capacity during post-match recovery.
